# Production and optimization of surfactin produced from locally isolated *Bacillus halotolerans* grown on agro-industrial wastes and its antimicrobial efficiency

**DOI:** 10.1186/s12866-024-03338-w

**Published:** 2024-06-03

**Authors:** Mohamed Abdelraof, Mohamed U. Nooman, Amr H. Hashem, Amr S. Al-kashef

**Affiliations:** 1 Microbial Chemistry Department, Biotechnology Research Institute, National Research Centre, Cairo, Dokki, 12622 Egypt; 2 Biochemistry Department, Biotechnology Research Institute, National Research Centre, Cairo, Dokki, 12622 Egypt; 3 Botany and Microbiology Department, Faculty of Science, Al-Azhar University, Cairo, 11884 Egypt

**Keywords:** Surfactin, *Bacillus halotolerans*, Optimization, Characterization, Antimicrobial.

## Abstract

**Introduction:**

Optimal exploitation of the huge amounts of agro-industrial residuals that are produced annually, which endangers the ecosystem and ultimately contributes to climate change, is one of the solutions available to produce value-added compounds.

**Aim and objectives:**

This study aimed at the economic production and optimization of surfactin. Therefore, the production was carried out by the microbial conversion of Potato Peel Waste (PPW) and Frying Oil Waste (FOW) utilizing locally isolated *Bacillus halotolerans.* Also, investigating its potential application as an antimicrobial agent towards some pathogenic strains.

**Results:**

Screening the bacterial isolates for surfactin production revealed that the strain with the highest yield (49 g/100 g substrate) and efficient oil displacement activity was genetically identified as *B. halotolerans*. The production process was then optimized utilizing Central Composite Design (CCD) resulting in the amelioration of yield by 11.4% (from 49 to 55.3 g/100 g substrate) and surface tension (ST) by 8.3% (from 36 to 33 mN/m) with a constant level of the critical micelle concentration (CMC) at 125 mg/L. Moreover, the physiochemical characterization studies of the produced surfactin by FTIR, ^1^H NMR, and LC–MS/MS proved the existence of a cyclic lipopeptide (surfactin). The investigations further showed a strong emulsification affinity for soybean and motor oil (E24 = 50%), as well as the ability to maintain the emulsion stable over a wide pH (4–10) and temperature (10–100 °C) range. Interestingly, surfactin had a broad-spectrum range of inhibition activity against *Bacillus subtilis, Staphylococcus aureus, Pseudomonas aeruginosa, klebsiella pneumonia*, and *Candida albicans.*

**Conclusion:**

Subsequently, the screening of the isolates and the utilized food-processing wastes along with the extraction technique resulted in a high yield of surfactin characterized by acceptable ST and CMC levels. However, optimization of the cultural conditions to improve the activity and productivity was achieved using Factor-At-A-Time (OFAT) and Central Composite Design (CCD). In contrast, surface activity recorded a maximum level of (33 mN/n) and productivity of 55.3 g/100 g substrate. The optimized surfactin had also the ability to maintain the stability of emulsions over a wide range of pH and temperature. Otherwise, the obtained results proved the promising efficiency of the surfactin against bacterial and fungal pathogens.

## Introduction

Microbial biosurfactants, low molecular weight surface-active substances with significant industrial value, are stable in severe environmental conditions and have favorable chemical characteristics [1]. One example of a low molecular weight amphiphilic lipopeptide biosurfactant is surfactin which has many industrial applications in agriculture, food, medicine, cosmetics, mining, petroleum, and environmental administration. Surfactin has environmental harmony due to its low toxicity, and biodegradability [2, 3]. Generally, surfactin is produced by microorganisms such as *Bacillus* species and acts as an antibiotic agent for a broad spectrum of human and plant pathogens [4]. Among the biosurfactant family, surfactin has the most effective activity toward surface tension reduction [5].

The choice of producer microbe, kind of substrate, and purification technique determines surfactin production cost [1]. Unfortunately, one of the biggest challenges facing the production of surfactants by microbial sources is to find a sustainable culture medium characterized as low-cost green eco-friendly to achieve large-scale industrial production. Since, synthetic medium used for production does not agree with the requirements of the industrial sector, due to its high cost, in addition to the challenge of acquiring medium components in significant quantities, and the environmental pollution caused by these chemicals [4]. Furthermore, the isolation of robust local strains also has an important impact on the reduction of production costs [6]. Therefore, alternative sources for the cultural media used as substrates regarding microbial surfactin production are considered potent trends in recent decades.

On the other hand, several food residuals are generated in our daily life with huge quantities exceeding hundreds of tons, causing extensive environmental pollution and economic loss. Regarding the highly consumed crops, fruits, vegetables, and dairy products in the food industries, agricultural waste is one of the most produced wastes around the world. Recycling agricultural waste by exploitation in microbial fermentation, not only decreases environmental pollution but also adds important value-promising products such as surfactin [4].

Accordingly, a large amount of potato peel waste (PPW) produced by plant-based food manufacturing is currently causing big environmental pollution. Around 69.5% of total produced potatoes are involved in industrial processes in developed countries, therefore, huge quantities resulted from the potato industries ranging from 15 to 40% of the product mass [6, 7]. The negative impact of the PPW has been treated by the improvement of management that led to an integrated recycling platform which able to benefit from these byproducts [7]. Moreover, oil waste from various vegetable oil refineries and industrial oil waste such as frying oil waste (FOW) in addition to other agricultural wastes, contribute to environmental pollution, causing climate change, a phenomenon that increases the environmental issues in this era [8].

Numerous researchers and industries have made a great effort to create fermentation industries utilizing agricultural wastes [9]. In this context, an association of PPW with FOW for the production of economic surface-active compounds was promising, as these waste combinations have extensively encouraged the microbial strain for the production of biosurfactants [6].

To our knowledge, there has been a scarcity of reports studying surfactin production by solid-state fermentation, particularly from *B. halotolerans* utilizing PPW and FOW as studied in our previous work [10]. Therefore, this study was designed to optimize the production process of the previously produced surfactin from *B. halotolerans* by the same agro-industrial wastes under solid-state fermentation. In addition, the emulsification activity studies and investigation of the antimicrobial activity of the produced surfactin against some microbial pathogens will be performed.

## Materials and methods

### Materials

The frying oil waste (FOW) and potato peel waste (PPW), were collected from potato processing factories, Giza, Egypt, and stored at − 4^o^C. The pathogenic strains that were used in the antimicrobial investigation were kindly donated by the culture collection of the Microbiology and Immunology Dep., Faculty of Medicine, Al-Azhar University, Cairo, Egypt. All chemicals and solvents were of analytical grade.

### Isolation of surfactin-producing bacteria

Collection of the soil and sludge samples from the oil-contaminated regions was carried out using sterilized bottles and stored in the refrigerator until used. Preparation of the nutrient agar medium supplemented with an antifungal agent (i.e. cyclohexamide at 50 mg/L) and platted under aseptic conditions was carried out [11]. Then, different samples were subjected to the serial dilution technique to justify the concentration of each sample to 10^4^ and 10^7^ dilutions and swapped over the prepared nutrient agar plate. The inoculated culture plates were incubated at 37 ^o^C for 48 h and the separated bacterial colonies were picked up and preserved using nutrient agar slants at 4 ^o^C for further studies. The mixed colonies were also subjected to further purification under the serial dilution method [12].

### Surfactin production medium and cultivation conditions

Screening of surfactin-producing ability for all bacterial isolates was subsequently evaluated under solid-state fermentation. Each bacterial isolate was pre-activated using a nutrient broth medium for 24 h under shaking conditions. In general, Agricultural residuals were considered as a preferred culture medium in terms of those reduces the environmental pollution and decreases the cost of culture medium. Therefore, POW (carbon source) and FOW (inducer source) were utilized as a main culture medium for cultivation of bacterial isolates. Moistened of the culture medium using the PPW filtrate supplemented with trace elements was performed and the inoculated culture medium was incubated at 37 ^o^C for 5 days under static conditions [6]. The capabilities of the bacterial isolates to secrete surfactin in the culture medium were assessed based on the oil displacement method (will be described later). The most powerful surfactin-producing bacterial isolate was then subjected to molecular identification.

### Molecular identification of most-potent isolated strain

After the determination of the most active biosurfactant producer isolate, identification using a molecular characterization technique based on the 16s rRNA was applied. In this way, genomic DNA isolation, subjected to the purification kit, and sequencing of the pure gene was achieved based on the standard producer characterized by Macrogen Company (Seoul, South Korea; https://www.macrogen.com). In brief, Amplification was done using forward primer 8 F (5′-CAG GCC TAA CAC ATG CAA GTC-3′) and reverse primer 1492R (5′-GGG CGG GGT GTACAA GGC-3′). The PCR mixture was carried out in a volume of 50 µl, containing 22 µl of MQ, 25 µl of DreamTaq Green DNA Polymerase (Thermo Fisher Scientific, USA), 1 µl of each forward and reverse primer (10 µmol/l), and 1 µl of template. The PCR amplification conditions were 4 min of preheating at 95 °C, 30 s denaturation at 95 °C, 45 s of primer annealing at 50 °C, 1 min extension step at 72 °C, and post cycling extension of 10 min at 72 °C for 35 cycles. The reactions were carried out in a thermal cycler (Applied Biosystem Thermal Cycler, USA). The amplicons were purified and then directly sequenced using the ABI 3730 DNA Analyzer (Applied Biosystems). The resulting sequences were aligned and compared with the related sequences deposited in GeneBank (http://blast.ncbi.nlm.nih.gov/Blast.cgi) using the Basic Local Alignment Search Tool (BLAST). Consequently, phylogenetic analysis of the obtained sequence was conducted using Molecular Evolutionary Genetic Analysis Software (MEGA version X), and the phylogenetic tree with the related strains was then constructed. The 16 S rDNA gene sequence of the bacterial strain used in this study has been deposited in the GeneBank nucleotide sequence database under the accession number MW715019, and the strain was identified as *Bacillus halotolerans*.

### Extraction of surfactin

According to the modified method described by [13], the surfactin compound was extracted by 100 mL methanol added to the produced culture, and then the mixture was agitated in a reciprocal shaker at 40 ℃ for 2 h and 160 rpm. The extracted compound was then filtered via filter paper Whatman No. 2. Finally, the solvent was evaporated by a rotary evaporator (heidolph cooling analog vacuum controller G1-Germany) at 40 ℃ to obtain the methanolic extract of *B. halotolerans*.

### Optimization process and statistical analysis

Enhancement of surfactin production could be related to the optimization of the nutritional and cultural parameters. Since these parameters considered a vital key that plays an important role in the progress of the production process. Therefore, the One-Factor-at-a-Time (OFAT) protocol was investigated for each condition to determine the significant ones. Thus, several cultural parameters were examined individually under solid-state fermentation (SSF) and the significant ones were selected for further investigation. Meanwhile, factors that not impacting on the production process were subsequently neglected. Accordingly, the independent variables, incubation period, and FOW concentration were found to be more effective on the surfactin production by *Bacillus halotolerans* and subjected to the Response Surface Methodology (RSM) approach using Central Composite Design (CCD).

### Central composite design (CCD)

To investigate the relationship between independent and dependent variables, RSM was used with one of the central composite design methods, a central composite face-centered (CCF) design. Development of the optimization curve was conducted using the Minitab® DoE statistical package’s response optimizer to identify the precise combinations of the interacting parameters that would enhance surfactin productivity [11]. In this study, independent factors incubation period and oil concentration were the factors affected surfactin production according to the OFAT method. The center points of these factors were selected according to the results of OFAT. Thirteen runs of s CCD contain incubation period and oil concentration at five levels for each factor as illustrated in (Table 1). Levels of period were 5, 7, 9, 11, and 13 days, while levels of inducer, oil concentration were 1, 2, 3, 4, and 5%. The determination of the model’s significance was based on an analysis of variance. The regression equation was derived, and a P value below 0.05 was used as the threshold to indicate the significance of the model term.

**Table 1:** The selected factors and levels for the optimization process

Factor	L1	L2	L3	L4	L5
Period (days)	5	7	9	11	13
Oil concentration (%)	1	2	3	4	5

The experimental design composed of 13 experimental runs was created; among these, five runs were carried out at the center point values, while each remaining run will apply at 5 levels by combinations of levels of both two parameters. In the CCD, two levels were utilized to determine whether the maximum production was obtained at lower or higher concentrations of the variables by comparing them with the experimental results obtained from center point values. The significance of the model was determined by analysis of variance, the regression equation was obtained, a P value less than 0.05 indicates that the model term is significant.

### Validation of the results

Following the theoretical optimization of the two independent parameters for reducing CMC % value by *B. halotolerans*, the response optimizer’s optimum conditions were experimentally implemented and compared to the expected outputs. The applications of the conditions were carried out in three replicates and the results were reported as mean ± standard deviation.

### Structural characterization of the produced surfactin

#### Fourier transform infrared spectroscopy (FTIR) analysis

The FTIR analysis was carried out by a Bruker VERTEX 80 (Germany) combined Platinum Diamond attenuated total reflectance, using a diamond disc at range of 4000–400 cm^− 1^ with a resolution of 4 cm^− 1^ at 2.4 refractive index.

### ^1^H NMR spectroscopy

The ^1^H NMR spectra analysis was performed by Varian Mercury VX-300 NMR spectrometer in deuterated chloroform (CDCl_3_) solvent. At 300 MHz, spectra were conducted.

### **LC-ESI-MS/MS analysis**

The produced surfactin LC-ESI-MS/MS analysis was accomplished utilizing liquid chromatography-electrospray ionization–tandem mass spectrometry (LC-ESI-MS/MS) SCIEX Triple Quad 5500 + MS/MS and ExionLC AC systems were used for the separation while, electrospray ionization (ESI) for detection. The separation was done by a Ascentis® C18 Column of 4.6 × 150 mm, 3 μm dimensions. Compound identification was performed using MS-DIAL software version 4.70 and Fiehn HILIC library.

### Functional characterization of produced surfactin

#### Oil displacement method

To perform the oil displacement test, 15 µl of crude oil was gently added to 40 ml of distilled water in a 15 cm-diameter Petri dish. The oil was then distributed over the water’s surface area. Aqueous solution (10 µl) containing surfactin (0.1% in H_2_O) was added to the oil layer’s surface. The clear zones that had formed were measured, recorded, and their average diameter was computed in triplicate as a percentage of the Petri dish’s diameter [14].

### Surface tension (ST) and critical Micelle concentration (CMC) estimations

The surface tension of 0.2% aqueous solution of the produced surfactin was assessed at 25 ℃, using the ring method by KrÜss Processor tensiometer-K100, Germany. While the CMC was estimated from the breakpoint in ST against concentrations of the produced surfactin. Each concentration was triplicated, and the average was reported according to [15].

### Emulsification studies of the produced surfactin

The emulsification activity is defined as the surfactin compound’s ability to maintain the structure of the emulsion over a definite period. According to the method described by [5], the optimized surfactin (33 mN/m) was prepared in a concentration of 0.2% in distilled water. The surfactin solutions (2 ml) were mixed with 2 ml of hydrocarbons (motor oil and hexane) or vegetable oils (corn and soybean oils) in a graduated test tube, then the mixture was shaken by vortex mixer (heidolph Reax top) at high speed for 2 min. for each emulsification index (E_24_) and the emulsion stability test. The emulsification index E_24_ (%), was calculated according to the following equation:

E_24_ (%) = height of the emulsion layer **/** total height of the mixture **×** 100.

However, the emulsion stability was determined during 7-day intervals.

### Stability studies against extreme environmental conditions

Stability studies were carried out for surfactin extract using soybean oil as follows. The temperature stability was examined by heating 2 ml of 50 mg/l in distilled water at 10, 25, 55, 70, and 100 °C for 15 min then left to settle down to room temperature, after which the emulsification index was measured. As for the stability of pH, 50 mg/l of surfactin extracts were prepared at different pH values (2, 4, 6, 8, and 10) using HCl or NaOH and then emulsification activities were assessed. The effect of salinity concentrations (2, 4, 6, 8, and 10 NaCl %) was also measured [16].

### Antimicrobial activity of *B. halotolerans* surfactin

Investigation of the antimicrobial activity of the produced surfactin was implemented using clinically important pathogens derived from the Microbiology and Immunology Dept., Faculty of Medicine (Boys), Al-Azhar University, such as Gram-positive bacteria (*Bacillus subtilis*, *Staphylococcus aureus)*, Gram-negative bacteria (*Pseudomonas aerginousea*, *E.coli*), and fungi (*Candida albicans, Aspergillus niger).* Preparation of each pathogen was conducted prior to any experiment under specific conditions. The bacterial preinoculum was incubated using a nutrient broth medium at 37 ^o^C for 24 h meanwhile, the fungal preinoculum was cultivated in Potato dextrose broth medium at 28 ^o^C for 48 h under shaking conditions. Accuratly, justification of inoculum size for each pathogen was carried out using Colony Forming Unite (CFU) in order to be constant throughout all performed tests.The agar well diffusion method was consequently applied to evaluate the efficiency of the surfactin to prevent microbial pathogen proliferation. Each tested pathogen justified its inoculum size based on Colony Forming Unite (CFU) to be closed to 10^− 6^ and dispersed over the Muller Hinton Agar medium. The addition of the surfactin compared to the standard antibiotic agents was conducted and all tested pathogens were incubated under specific conditions for bacterial and fungal pathogens [17, 18]. The targeted surfactin was investigated at a fixed concentration (20 µg/mL) in comparison to the reference antibacterial and antifungal agents such as Cephradine, Ciprofloxacin, Fluconazole, and Amphotrecine B. The activity of the surfactin was evaluated based on the inhition zone diameter (mm) that obtained around each microbial pathogen.

### Statistical analyses

The experimental results provided in this study were analyzed as the average ± standard deviation (SD) for *n* = 3 using standard analysis of Student’s t-test.

## Results and discussion

One of the crucial elements that contribute to lowering the cost of the manufacturing process is the isolation of microorganisms from their surrounding environment; additionally, selecting the robust one that can provide a higher yield with favorable quality of the desired products [19]. Isolation of bacterial colonies was carried out using a nutrient agar medium supplemented with an antifungal agent, different pure isolates were picked up according to the morphological and microscopically properties. Preparation of each isolate initially occurred using a nutrient broth medium at 37 ^o^C for 24 h under shaking conditions.

### Screening and production of surfactin by the bacterial isolates

**Fig. 1:**
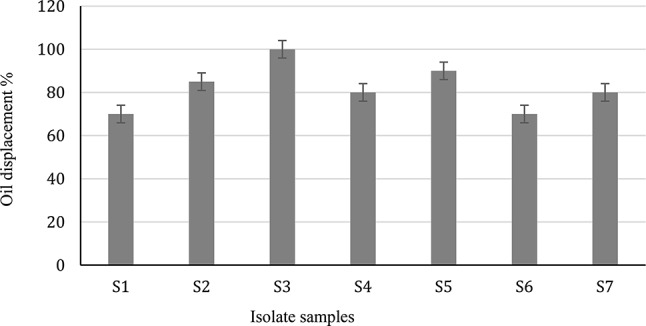
The screening of the produced compounds toward surface tension activity by oil displacement test and productivity of the bacterial different isolates (S1 - S7)

Screening of each bacterial isolate to investigate its ability to secrete surfactin was conducted under the SSF technique utilizing the PPW and FOW, as agricultural byproducts. The new culture medium encouraged the bacterial isolates to produce surface-active compounds. Moreover, the methanol extraction method showed a variation in the activity of surfactin produced by the bacterial isolates. In this respect, according to the oil displacement method, the potent activity of the surfactin was related to the isolate coded as S3, followed by S5, S2 then S4 and S7, since the maximum oil displacement activity of the produced surfactin (100%) was related to the S3 sample (Fig. 1). This was followed by the isolate coded as S5 (90%), while S2 gave the activity of 85%. The S4 and S7 provided 80% activity, and finally, isolate samples S1 and S6 contributed the lowest activity (70%). These results indicated the superiority of isolate S3 during the screening test, whereas the S3 methanol extract, achieved the highest productivity (49.1 g/100 g substrate) associated with the highest activity (100%). Therefore, the encoded isolate S3 was chosen for further investigation in this study.

### Identification of S3 isolate by genetic characterization

**Fig. 2:**
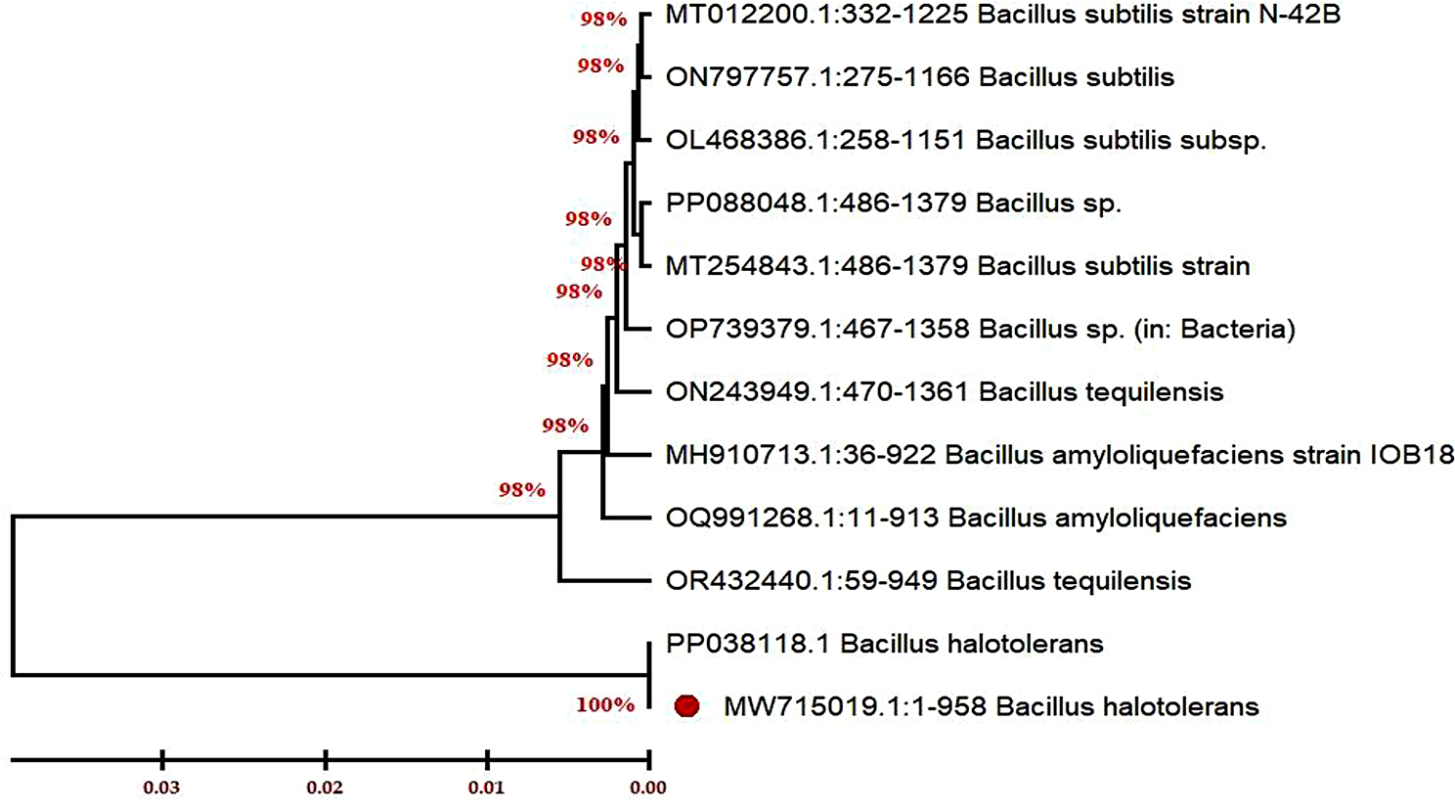
Phylogenetic tree of the surfactin-producing isolate

Selection of the most potent bacterial isolate (i.e. S3) after the screening examination was carried out and then subjected to genetic identification using the 16s rDNA method. Extraction of the total genomic DNA was firstly achieved followed by the PCR amplification of the specific gene 16s rDNA using a universal primer as mentioned in the material and methods section. Subsequently, the PCR product was purified from any impurities or primer dimer that appeared after running the samples using the gel electrophoresis method. Finally, the purified sample was sequenced and then analyzed in the GeneBank library using BLAST (http://blast.ncbi.nlm.nih.gov/Blast.cgi*).* Relatedness studies of the complete targeted sequence of the 16s rDNA for this isolate definitely belonging to the *Bacillus* species, about more than 99% of similarity. In addition, the construct of the phylogenetic tree was also designed using MEGA X (Fig. 2), which besides the morphological observations clearly showed this isolate corresponded to *Bacillus halotolerans* and thus was recorded in the GeneBank under accession number, MW715019.

### Statistical optimization analysis of surfactin production medium

An extensive investigation was carried out on the surfactin production by several *bacillus* species; however, there were a few studies that were interested in the production using a low-cost culture medium under solid-state conditions and rare ones utilizing solid-state fermentation techniques for surfactin production. Therefore, exploitation of the food-processing waste in the production process provides a promising method that can be displayed as a significant candidate in different industrial sectors [7].

Enhancement of the surfactin production by *Bacillus halotolerans* is the more excited target to obtain it in maximum activity and productivity. Therefore, optimization of the cultural and nutritional conditions was first implemented using the Factor-At-A-Time (OFAT) procedure. Seven independent parameters were applied as follows, PPW content (20–50 g/L), Oil concentration (2- 5%), temperature (25–35 ^o^C), pH (5–7), Moisture content (50- 70%) and incubation period (3–9 days) to determine the significant ones. A plausible increase in surfactin activity and productivity was noticed when investigated using different Oil concentrations and incubation period conditions. Since the CMC % was remarkably decreased when the oil concentration and incubation period were increased (Data not shown). Otherwise, the other parameters do not affect the surfactin activity or productivity, which does not have any decreases in the CMC % level. Therefore, the promising parameters, oil concentration, and incubation period, the impactful parameters were then applied via advanced statistical programs such as Central Composite Design (CCD).

In this regard, the production of surfactin by *Bacillus* species was extensively reported by many authors, particularly using a synthetic culture medium under submerged fermentation. Therefore, the valorization of the agricultural byproducts could be contributed to the efficiency of the production process in terms of yield and cost. The exploitation of the food-processing byproducts considerably impacted biosurfactant production by *Bacillus subtilis* #573 using corn steep liquor (CSL) as a culture medium [20]. Potato peels also had a major starchy zero-value food residual, mainly composed of 80% water, 17% carbohydrates along with other minor components, thus valorized of potato peel effluent was used as a culture medium for the production of surfactin by *Bacillus subtilis* [21].

### Statistical optimization of surfactin production using CCD

**Fig. 3:**
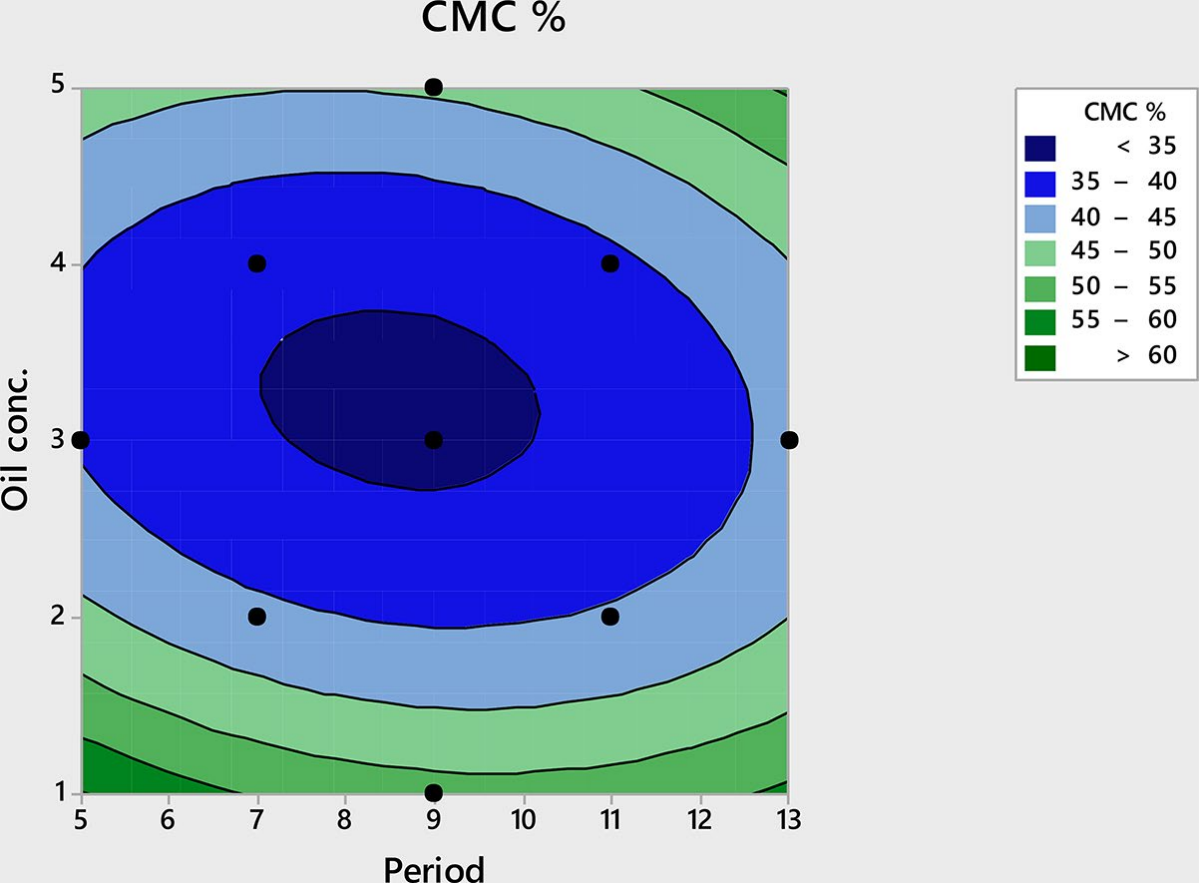
Contour plots of factors affecting surfactin production

To further optimize the most significant factors (oil concentration and incubation period), CCD was designed for this purpose. In this way, a combination between the two significant parameters was designed and other independent variables in the culture medium would be constant, which facilitates the investigation of the interplay between them. In this way, CMC % was assessed for each run and compared with predicted results after data analysis (Table 2). Noticeably, the practical results for all runs were relatively consistence with the predicted results. Furthermore, the decrease in CMC % value was enhanced through different run conditions, which illustrated a positive response toward the designed CCD. As can be seen in (Fig. 3), the contour plot showed a good interaction between the Oil concentration and incubation period, and a significant elliptical shape for the contour plot was clearly observed.

On the other hand, analysis of variance (ANOVA) was determined based on the CMC % results. P values are used as a statistical measure to assess the significance of individual coefficients. These coefficients are crucial for comprehending the underlying patterns of mutual interactions among the variables under examination. A significance level of less than 0.05 indicates that the model terms are statistically significant. In addition, the Model F-value of 18.33 indicates the significance of the model. The ANOVA results for the fitting of the quadratic model are presented in (Table 3). This table illustrates the model is highly significant where P-value was 0.001. According to linear parameters, oil concentration was highly significant where the P-value was 0.025, while as period was non-significant where the P-value was 0.481. Furthermore, square parameters A*A & B*B exhibited significant effects where the P-value was 0.007 and 0.001 respectively. Moreover, the observed “Pred R-Squared” value of 92.90% exhibits a substantial level of concordance with the corresponding “Adj R-Squared” value of 87.83%. Furthermore, the R2 refers to a potent fit between the predicted and measured values of the produced surfactin, which emphasizes that the model is significant for this process. The R2 value is one of the most important indicators that clearly demonstrates the trustworthiness of the statistical program. Since the accuracy of the model is relatively correlated with the increase of the R2 value to 100.

Multiple regression analysis of the experimental results provides the second-order polynomial equation for surfactin production in terms of uncoded factors is shown as follow: CMC% = (110.9–7.75 A − 26.82 B + 0.380 A*A + 3.644 B*B + 0.375 A*B) Where A is incubation period, B is oil concentration.

**Table 2:** CCD for improvement production of SL

Run order	Incubation Period (day.)	Oil conc.	CMC%	Predicted CMC %
1	11	4	41	39.0575
2	7	4	40	36.7241
3	9	1	52	52.0546
4	9	5	44	45.7213
5	13	3	41	41.2213
6	9	3	34	34.3103
7	9	3	34	34.3103
8	11	2	41	40.7241
9	9	3	33	34.3103
10	9	3	34	34.3103
11	7	2	43	41.3908
12	5	3	38	39.5546
13	9	3	33	34.3103

**Table 3:** Analysis of Variance (ANOVA)

Source	DF	Adj SS	Adj MS	F-Value	*P*-Value
**Model**	5	344.598	68.920	18.33	0.001
**Linear**	2	32.167	16.083	4.28	0.061
A	1	2.083	2.083	0.55	0.481
B	1	30.083	30.083	8.00	0.025
**Square**	2	310.182	155.091	41.24	0.000
A*A	1	52.898	52.898	14.07	0.007
B*B	1	304.330	304.330	80.92	0.001
**2-Way Interaction**	1	2.250	2.250	0.60	0.465
A*B	1	2.250	2.250	0.60	0.465
**Error**	7	26.325	3.761		
Lack-of-Fit	3	25.125	8.375	27.92	0.004
Pure Error	4	1.200	0.300		
**Total**	12	370.923			

A and B mean period and oil concentration respectively

### Validation of the results

**Fig. 4:**
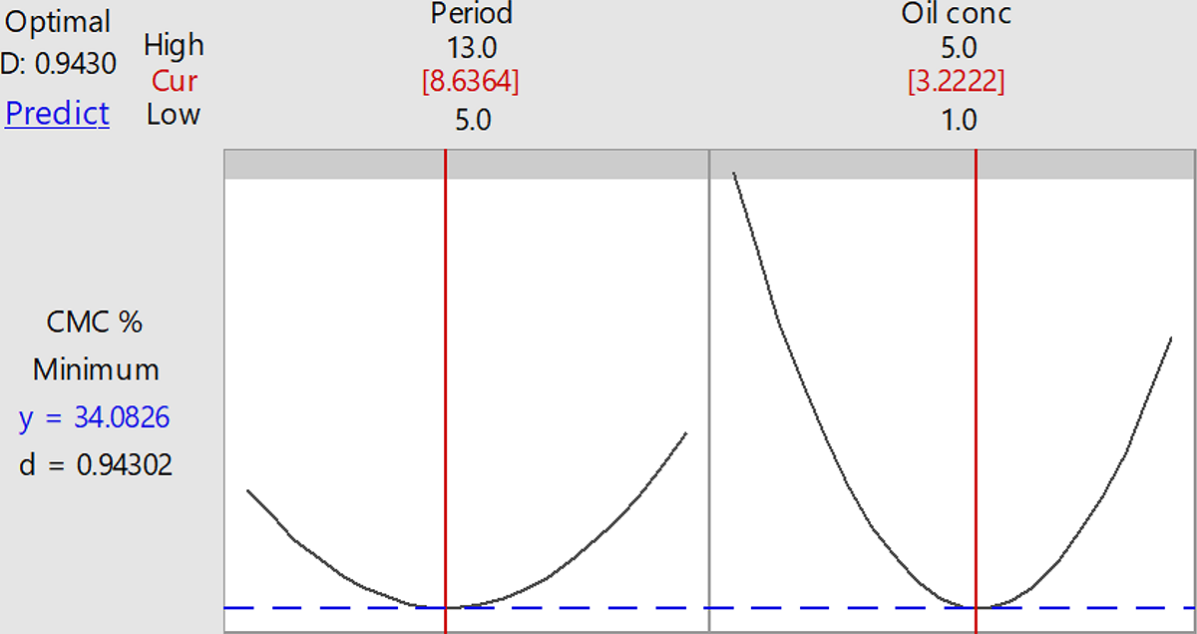
Response optimizer for surfactin production by *B. halotolerans*

The successful performance of the designated program was confirmed by examining a predicted condition that was extracted after analysis of the results. Therefore, to confirm the optimized conditions, three different conditions with the desirability of (1.0) and minimum ST value were determined (Fig. 4). In, the final optimal values for the significant parameters were found to be close to the predicted result (34.08826%). Hence, the agreement of the practical CMC % value with the practical result refers to the validity of the designated model. The validation of the observed CMC % value demonstrated an average confidence of 94.71% and a minimum surfactin value (35.8%). Accordingly, the optimized conditions that were able to provide a considerable enhancement in the surfactin activity (35.8%) were as follows, 3.222% of the oil concentration and 8.6364 of the incubation period, which was nearing the predicted value (34.08826%).

Waste oils extracted in large quantities from food and industrial sources commonly cause severe environmental pollution. However, the valorization of these oil wastes via conversion to valuable products without releasing any harmful substances displayed an important purpose in terms of economic and ecological considerations. Bioconversion of waste oils to biosurfactant by microorganisms is one of the important green applications that play a vital role in disposing of this waste safely. In addition, oil wastes are a potent inducer for biosurfactant production, which could be incorporated in the synthesis of the intermediate components that achieve the biosurfactant pathway [4]. Biovalorization of the FOW was successfully converted to different valuable compounds by several microorganisms [22]. Frying Oil Waste (FOW) was found as an important enhancer for the surfactin production by *Bacillus halotolerans* in this report. This contributed to the improvement of the surfactin yield and the CMC % was gradually decreased. In accordance with the obtained results, a maximum oil displacement area along with the lowest surface tension of the surfactin was obtained from *Bacillus* sp. HIP3 when cultivated in 2% of FOW for 7 days [23]. Moreover, investigating the ability of some *bacillus* species to produce surfactin using emulsified FOW was also could be of interest for the production of pure surfactin, particularly by *Bacillus subtilis* [24]. In addition, the utilization of different oils to enhance the biosurfactant production by bacterial cells was frequently performed; surfactin production by *Bacillus subtilis* LSFM-05 (230 mg/L of the purified biosurfactant) was carried out using raw glycerol, obtained from a vegetable oil, as the sole carbon source was carried out by [25]. Crude glycerol has also been able to display a low-cost and inducer carbon source for the surfactin production (2.8 g/L) by *Bacillus subtilis* #309 [5].

The extraction technique and optimization process of surfactin, increased the yield by 11.4% (from 49.1 to 55.3 g/100 g substrate) ST by 8.3% (from 36 to 33 mN/m) with a constant level of the CMC at 125 mg/L. Given the reports’ rarity studying the production of surfactin from Bacillus spp. by solid fermentation limited the yield of the produced surfactin to 3.07 and 7.4 g/100 g substrate [26, 27]. Therefore, this may support the solid-state fermentation technique and methanol extraction for surfactin production, which could reveal a concept for increasing the yield from Bacillus spp.

### Structural characterization of the produced *B. halotolerans* surfactin

#### **Fourier transform infrared spectroscopy (FTIR) of B. halotolerans surfactin**

The FTIR analysis of the produced surfactin was previously performed and the results indicated the existence of alkanes (C–H) and ester carbonyl group (C = O), from the peptide group in the produced compound. The stretching of C-N aliphatic amines and, NH-stretching were also presented which indicated the existence of peptides. The aliphatic amino acid chains appeared, in which results suggest the presence of lipopeptide in the extracted compound. These results pointed out that the produced biosurfactant by *B. halotolerans* is a cyclic lipopeptide type of biosurfactant (surfactin) as mentioned in our previous work [10].

#### ^1^H NMR spectra analysis of B. halotolerans surfactin

The analysis by ^1^H-NMR spectrum for the *B. halotolerans* extracted compound was previously reported [10] whereas the results confirmed the exitance of CH_2_ groups of aliphatic chains and, the group of CHO ester. The amino acids CH group existence may be considered a cyclic lipopeptide.

#### LC-MS/MS analysis of B. halotolerans surfactin

The liquid chromatography-mass spectrometry (LC-ESI-MS/MS) analyses for the extracted biosurfactant was also carried out previously [10]. The fragments that appeared from the analysis suggested the presence of a cyclic lipopeptide (7 amino acids, leucine, or isoleucine) with a fatty acid side chain of C12. However, the peaks in the analyses confirmed that these fragments were related to a lipopeptide consisting of 7 amino acids namely leucine or isoleucine with the fatty acid side chain of C14. The characterization studies however, of the produced surfactin, by FT-IR, ^1^H NMR, and LC-ESI-MS/MS analyses previously confirmed the existence of cyclic lipopeptide (surfactin) produced by *B. halotolerans.*

#### Surface tension (ST) measurement and CMC properties of surfactin

**Fig. 5:**
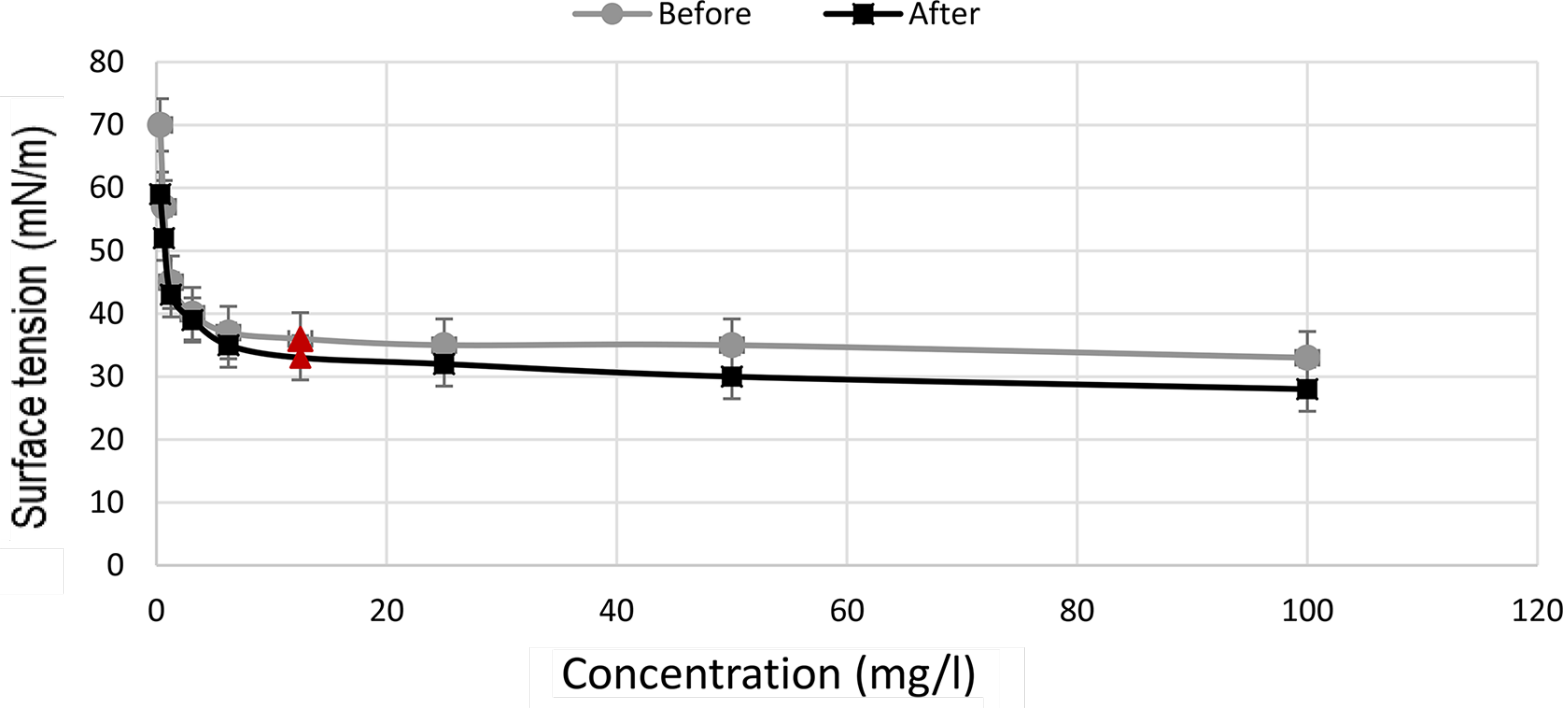
The surface tension vs. concentration and the critical micelle concentration (CMC) of the produced surfactin before and after optimization The CMC points are marked as triangles

Surface activities of the produced surfactin can be defined as the minimum concentration that is needed to reduce the ST to the maximum level (CMC). The produced surfactin, in this study, reduced the ST from 72 to 36 mN/m at the CMC of 125 mg/l before optimization [10] and 33 mN/m at the same CMC level after optimization process (Fig. 5).

#### Emulsification studies for the produced surfactin

**Fig. 6:**
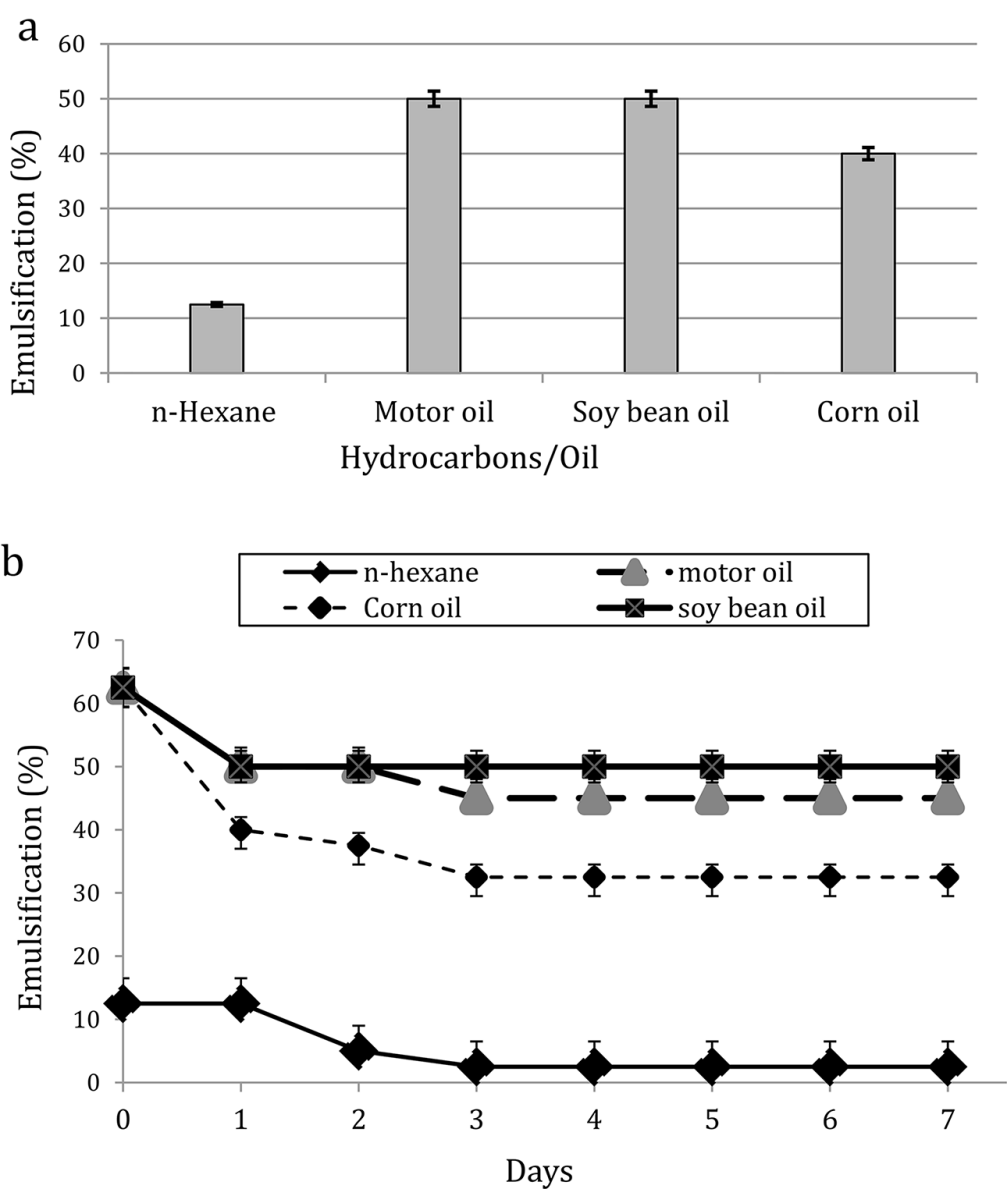
Emulsification efficiency (**a**) and emulsification stability (**b**) toward different hydrocarbons and vegetable oils with *B. halotolerans* surfactin, for 7 days at room temperature. Data were expressed as mean ± S.E. of 3 experiments

At the industrial scale, the stability of oil/water emulsion is crucial for the compound potential applications. Therefore, the emulsifying activity of the produced surfactin as an emulsifier for water against various short and long-chain hydrocarbon substrates at 25 ° C was carried out (Fig. 6a). The results indicated a considerable emulsification activity of the produced surfactin, towards long-chain hydrocarbons, where the highest emulsification index was related to soybean and motor oils (E24 = 50%), followed by corn oil (E24 = 40%). However, the lowest emulsification index (E24 = 12.5%) corresponded to the short-chain hydrocarbon (n-hexane). The efficiency of emulsifiers against long-chain hydrocarbon, in fact, indicates their suitability for use in industrial applications [28]. found that the surfactin produced by *B. subtilis* grown on okara formed acceptable emulsion activities with motor and sunflower oils while, it failed to form emulsions with hexane, hexadecane, and soybean oil [29]. reported also, that the surfactin produced by *B. subtilis* established a considerable emulsification index with crude oil, kerosene, octane, and cetane against water, where their result was similar to obtained motor oil emulsification index (E24 = 50%) with crude oil (E24 = 56%).

#### Emulsification stability studies

The emulsification stability of the produced surfactin with various water-immiscible substrates (long and short-chain hydrocarbons) during 7 days is shown in (Fig. 6b). Findings have demonstrated that the produced surfactin formed stable emulsions with soybean oil (50%), motor oil (45%), and corn oil (32.5%) over a period of seven days. However, it was unable to generate a stable emulsion with short-chain hydrocarbon (n-hexane). Similar results were obtained [28] related to surfactin produced by *B. subtilis* for motor oil where the produced emulsion was stable for 7 days and also, no stable emulsion was observed with hexane. Conversely, their analysis did not reveal any stable emulsion for soybean oil.

#### Emulsification stability against extreme conditions

**Fig. 7:**
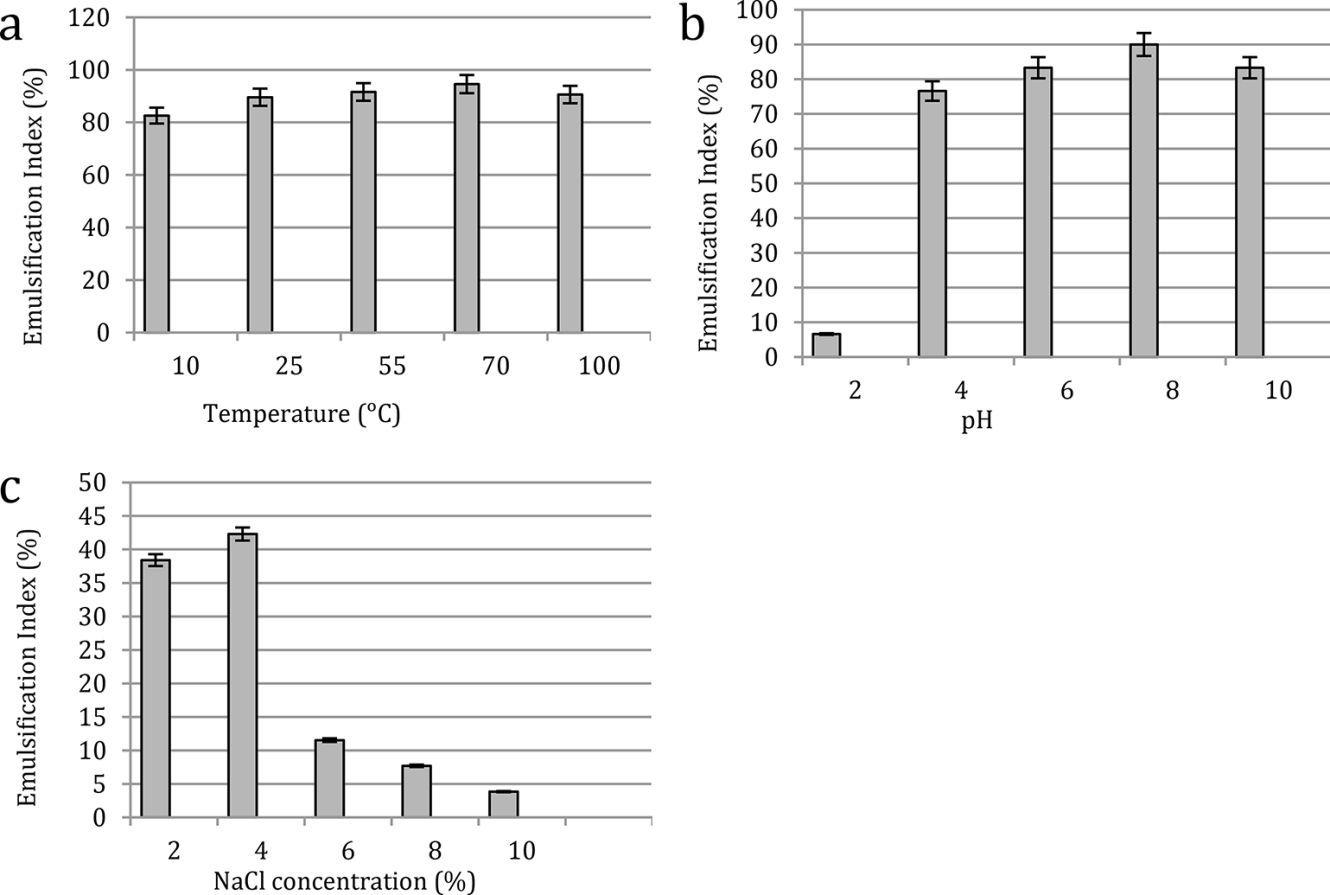
Stability of the produced surfactin at various (**a**) temperatures, (**b**) pH values, and (**c**) salinity concentrations

Emulsification activities have been proven to be strongly impacted by industrial factors or environmental tolerance, such as temperature, pH, and salinity. As a consequence, this study is very significant since it may restrict or expand the use of separated biomolecules in the future for various purposes. The produced surfactin emulsification index values were investigated against temperature, pH, and salinity (Fig. 7a, b, **and c**). With stability against heat treatment from 10 to 100 °C, the results of the emulsification index percentages remain at a considerably high level (82.6–94.6%), with the highest percentage (94.6%) corresponding to 70 ℃. Findings also indicated the effect of pH on the emulsifying activities as the formed emulsions maintain their high levels (76.6–90%) over a wide range of pH (4–10) where the maximum level related to pH 8 while, the lowest (6.6%) associated to the pH 2. However, salinity impact on the emulsification index showed that the obtained emulsions were unsuitable for use in high saline conditions with 6–10% NaCl. Yet, emulsification index percent were relatively high at NaCl concentrations of 2 and 4% with activity levels of 38.4 and 42.3%, respectively.

Based on the obtained results regarding the emulsification stability against extreme conditions, the produced surfactin was able to form stable emulsions tolerating high industrial temperature systems as well as acidic and alkaline conditions with an acceptable range of ionic stress (salinity), suggesting the tendency of the product to be applied industrially.

Ali et al. [30], investigated 4 bacterial strains, *Bacillus, Burkholderia, Providencia, and Klebsiella* biosurfactants against extreme conditions namely temperature, pH, and salinity. They found that all isolate biosurfactants reserved a constant level at low and high temperatures (4–100 °C). The emulsions of produced compounds also tolerated pH 2–10 while, *Bacillus* strain biosurfactant emulsion collapsed below 3% NaCl and increased gradually by higher concentrations. However, *Burkholderia*, *Providencia*, and *Klebsiella* biosurfactants emulsification activities showed similar stable emulsions (2–7% NaCl). Earlier, Pathak and Keharia [31] stated that *B. subtilis* K1 biosurfactant emulsification activity remained stable at 100 °C for 2 h, over a pH range of 6–12 h and up to 10% NaCl.

### Antimicrobial efficiency of the produced surfactin

**Fig. 8:**
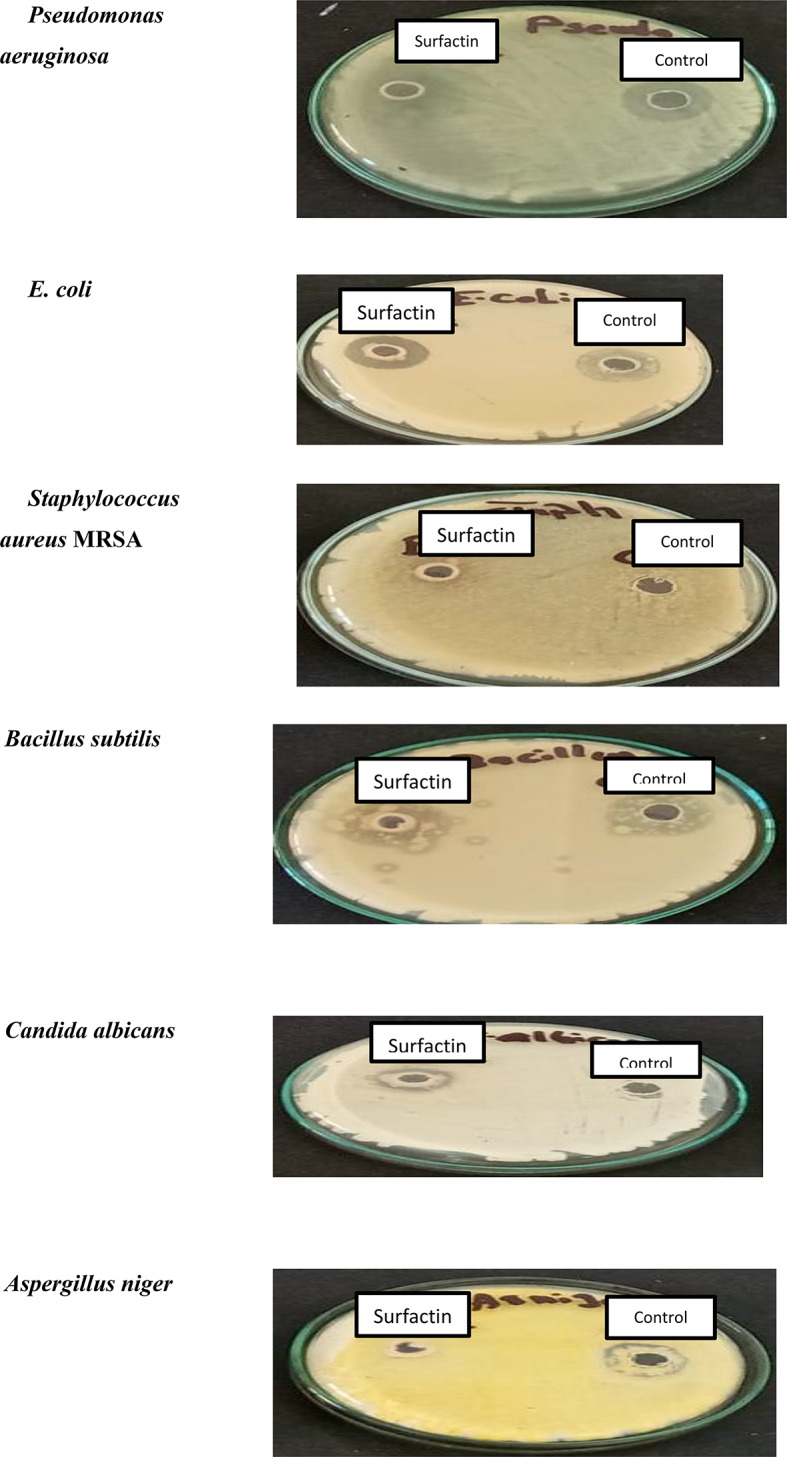
Antimicrobial activity of the targeted molecules using agar-well diffusion

The ability of the optimized surfactin to inhibit the proliferation of microbial pathogens was subsequently evaluated using the agar well diffusion method. In this regard, 20 µg/mL of each of surfactin and reference antibiotic agent were added to the inoculated plate and the inhibition ratio was investigated according to the inhibition zone (mm). As indicated in (Fig. 8), the superiority of surfactin was clearly observed towards all pathogens except for *Aspergillus niger*, which could proliferate in the presence of surfactin, while it was inhibited by the standard antifungal agent (i.e. fluconazole). Meanwhile, a clear killing effect of the surfactin was indicated against *Candida albicans* (3 mm), which resists the inhibition effect of the standard antifungal agent. As shown in (Table 4), a significant eradication activity of surfactin against each of *Pseudomonas aeruginosa* (4 mm), *E. coli* (5 mm), and *Bacillus subtilis* (4 mm) was achieved, which was found either equal or superior to that carried out by the standard antibacterial agent, ciprofloxacin. Furthermore, methicillin-resistance *Staphylococcus aureus* was slightly inhibited by the produced surfactin. A resistance of the bacterial pathogens towards a cephalosporin antibiotic group like Cephradine was reflected in their ability to degrade a β-lactam group, thus those pathogens having the ability to grow in the presence of this group of antibiotics.

**Table 4:** Antimicrobial activity of the targeted molecules using agar-well diffusion

Sample	Inhibition Zone (mm)					
	Candida albicans	Aspergillus niger	Bacillus subtilis	Staphylococcus aureus	E. coli	Pseudomonas aeruginosa
Surfactin	3	ND	4	2	5	4
Fluconazole ^a^	ND	3	-			
Amphotericin B ^a^	2	4	-			
Ciprofloxacin ^b^	-		3	2	4	3
Cephradine ^b^	-		ND	ND	ND	ND

a Fluconazole and Amphotericin were used as standard antifungal agents at 20 µg/ mL

b Ciprofloxacin and cephradine were used as standard antibacterial agents at 20 µg/mL,

cND: not determined

The antimicrobial activity of surfactin is extensively reported by many authors, which could prevent the proliferation of bacterial and fungal cells. The obtained results revealed the strong antimicrobial activity of the produced surfactin, which may be discussed by some possibilities, the cell membrane disintegration or inhibition of the protein synthesis or affecting the enzymatic activities of the microbial cells [32]. In agreement with our findings, a significant activity of produced surfactin toward *Staphylococcus aureus* strains, particularly *S. aureus* MRSA [33]. In addition, surfactin by *Bacillus subtilis* was found to be strongly active toward *Brachyspira hyodysenteriae* and *Clostridium perfringens* [34]. The antifungal activity of surfactin was also remarked against several fungal pathogens such as *Fusarium moniliforme*, which inhibited and damaged the hyphae of *F. moniliforme* in vitro [35].

## Conclusion

In this study, screening of different bacterial strains that were isolated from a contaminated environment was established to evaluate their ability to secrete novel surfactin under solid-state fermentation using agro-industrial wastes (PPW and FOW). A most potent bacterial producer, S3 was known as *Bacillus halotolerans* according to the genetic identification gave the highest yield attributed to the highest surface activity as well. the solid-state fermentation and methanol extraction of surfactin employed in this work revealed a new concept for increasing the yield of the produced surfactin from Bacillus species. The optimization, however, enhances the productivity from 49 to 55 g/ 100 g substrate (11.4%) and surface activity from 36 to 33 mN/m (8.3%) levels of the produced surfactin. The produced compound was found to have the ability to form stable emulsions with soybean, motor, and corn oils over a period of seven days. However, the produced emulsions were stable in extreme environmental conditions, tolerating high industrial temperature systems as well as acidic and alkaline conditions with an acceptable range of salinity. The surfactin was found to have a significant eradication activity against all tested pathogens except for *Aspergillus niger.*

## Data Availability

“The datasets generated and/or analysed during the current study are available in the NCBI repository, The 16S rDNA gene sequence of the bacterial strain used in this study has been deposited in the GeneBank nucleotide sequence database under the accession number MW715019, and the strain was identified as Bacillus halotolerans.

## Abbreviations

FOW: Frying Oil Waste
PPW: Potato Peel Waste
CCD: Central Composite Design
